# Targeted interference of SIN3A-TGIF1 function by SID decoy treatment inhibits Wnt signaling and invasion in triple negative breast cancer cells

**DOI:** 10.18632/oncotarget.11381

**Published:** 2016-08-19

**Authors:** Yeon-Jin Kwon, Boris A. Leibovitch, Nidhi Bansal, Lutecia Pereira, Chi-Yeh Chung, Edgardo V. Ariztia, Arthur Zelent, Eduardo F. Farias, Samuel Waxman

**Affiliations:** ^1^ Icahn School of Medicine at Mount Sinai, The Tisch Cancer Institute, New York, NY, USA; ^2^ University of Miami, Sylvester Comprehensive Cancer Center, Florida MI, USA

**Keywords:** triple negative breast cancer, invasion, SIN3A, TGIF1, Wnt, metastasis

## Abstract

Cancer cell invasion is an obligatory step for metastatic dissemination that contributes to rapid relapse and a poorer survival in triple negative breast cancer (TNBC) patients. Development of novel therapeutic strategies to block tumor invasion is an unmet need in the treatment of cancer. We reported that the selective inhibition of the PAH2 domain of SIN3A protein function markedly suppressed metastatic dissemination to the lungs in TNBC xenograft bearing mice. Here, we show that TNBC cell lines treated with Sin3 interaction domain (SID) decoy peptides that bind to PAH2 display a strong *in vitro* inhibition of transwell invasion. This is accompanied by actin cytoskeleton reorganization with increased cortical actin deposition and downregulation of known Wnt target genes that are associated with epithelial to mesenchymal transition (EMT) and cancer cell invasion. Wnt pathway inhibition by SID decoy peptide was confirmed by decreased Wnt reporter activity and altered cytoplasmic localization of nuclear β-catenin. TGIF1, a transcription factor that modulates Wnt signaling and known to interact with the PAH2 domain of SIN3A, can be dissociated from the SIN3A complex by SID decoys. TGIF1 knockdown inhibits WNT target genes and *in vitro* cell invasion suggesting that TGIF1 might be a key target of the SID decoys to block tumor invasion. Taken together, targeting SIN3 function using SID decoys is a novel strategy to reverse invasion and the EMT program in TNBC translating into the inhibition of metastasis dissemination and eradication of residual disease.

## INTRODUCTION

Triple negative breast cancer (TNBC) is an aggressive breast cancer subtype with high incidence in young African American women (∼30 years old), or patients with BRCA1/BRCA2 mutation [1–5]. The lack of expression of estrogen and progesterone receptors (ER and PR) along with no overexpression of epidermal growth factor receptor 2 (Her2) renders TNBC resistant to hormonal and other targeted therapies. Conventional chemotherapy is the standard of care with modest survival benefits. Moreover, the poor prognosis in TNBC is attributed to the high incidence of disseminated tumor cells leading to onset of metastatic disease and associated morbidity [6]. To overcome this clinical problem, there is an urgent need to not only identify drug targets but also to develop drugs that are effective in treating metastatic TNBC.

Our previous work has established the role of Sin3 paralogues (SIN3A and SIN3B) in TNBC pathogenesis [7–9]. Without intrinsic DNA binding capacity, Sin3 proteins interact with transcription factors and chromatin regulators via four paired amphipathic α-helix (PAH1-PAH4) motif to function as molecular scaffolds [7]. We have developed peptides and small molecule inhibitors (SID decoys) that block interactions between the PAH2 domain of Sin3 and set of chromatin-associated factors containing a conserved Sin3-interaction domain (SID) [7, 10–12]. Treatment of TNBC *in vitro* or *in vivo* with SID decoys promotes differentiation and luminal phenotype coincident with inhibition of epithelial-mesenchymal transition (EMT), cancer stem cells (CSCs) and distant metastasis [11, 12].

Underlying these strong anti-tumor effects, SID decoys inhibit key oncogenic signaling including the Wnt pathway. Wnt signaling is hyperactivated in 40-60% of TNBCs through strong expression of nuclear β-catenin and its targets [13, 14] and indicative of a poorer survival [15, 16]. Elevated Wnt/β-catenin activity promotes EMT, invasion and metastasis of breast cancer cells through activation of its downstream mediators (e.g. Axin2, BCL-9) [13, 16–18].

Here we report that SID decoy mediated inhibition of invasion and Wnt/β-catenin signaling of TNBC can result from the disruption of protein-protein interaction between the PAH2 domain of SIN3A and SID containing transcription factor TGIF1. Our novel findings identify SIN3A-TGIF1 protein interaction as a potential therapeutic target for preventing and treating metastases in patients with TNBC.

## RESULTS

### SID peptide inhibits invasion and induces cytoskeletal reorganization in TNBC cells

**Figure 1:**
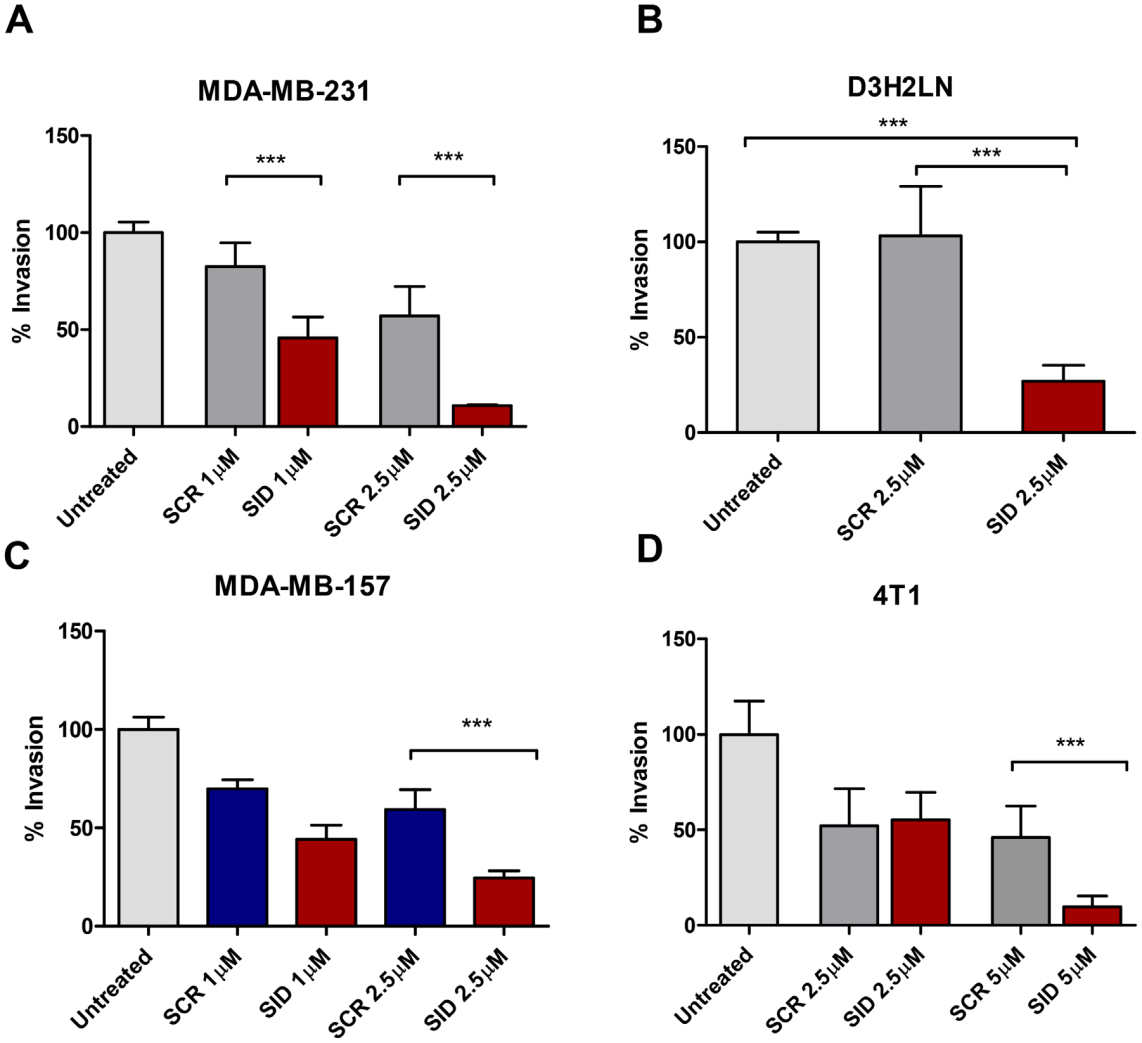
Treatment with SID decoy peptide inhibits transwell invasion of TNBC cells **A-D.** Transwell invasion assays were performed with MDA-MB-231 (A), D3H2LN (B), MDA-MB-157 (C), or 4T1 (D) cells that were either untreated (UT) or treated with SCR or SID (2.5μM) for 24 hours. The percentage of invasion for UT, SCR, and SID peptide treated cells was calculated by normalizing the number of cells invaded per filter to those in untreated control. (A; ^***^P<0.0001, B; ^***^P=0.0024, C, D; ^***^P<0.0001, One- way ANOVA and Tukey’s multiple comparison test, error bars, mean ± SEM).

We have previously shown that stable expression of SID peptide markedly inhibits growth of invasive TNBC colonies in 3-demensional (3D) Matrigel™ [10, 12]. Consistent with this, compared to the scrambled peptide (SCR), 24 h treatment of MDA-MB-231 with 2.5 μM SID peptide resulted in 81% inhibition of trans-well invasion in MDA-MB-231 cells (Figure 1A). Similar results were obtained in two additional human TNBC lines wherein 74% and 59% inhibition was observed in D3H2LN (a variant of MDA-MB-231 cells with a greater metastatic potential in comparison to parental MDA-MB-231 cells [19] (Figure 1B) and in MDA-MB-157 (Figure 1C) cells, respectively. SID peptide also blocked invasion in mouse 4T1 cells by 79%, albeit at a higher dose of 5 μM (Figure 1D). We also tested the effects of SID peptide on cell migration. Treatment of TNBC cells with SID peptide (1-2.5 μM) suppressed transwell migration in dose-dependent manner by 74% in MDA-MB-231 cells (Figure S1A), but not in MDA-MB-157, D3H2LN and 4T1 cells (Figure S1B-S1D). This suggests that unlike invasion, effect of SID peptide on cell migration is cell-line specific.

**Figure 2:**
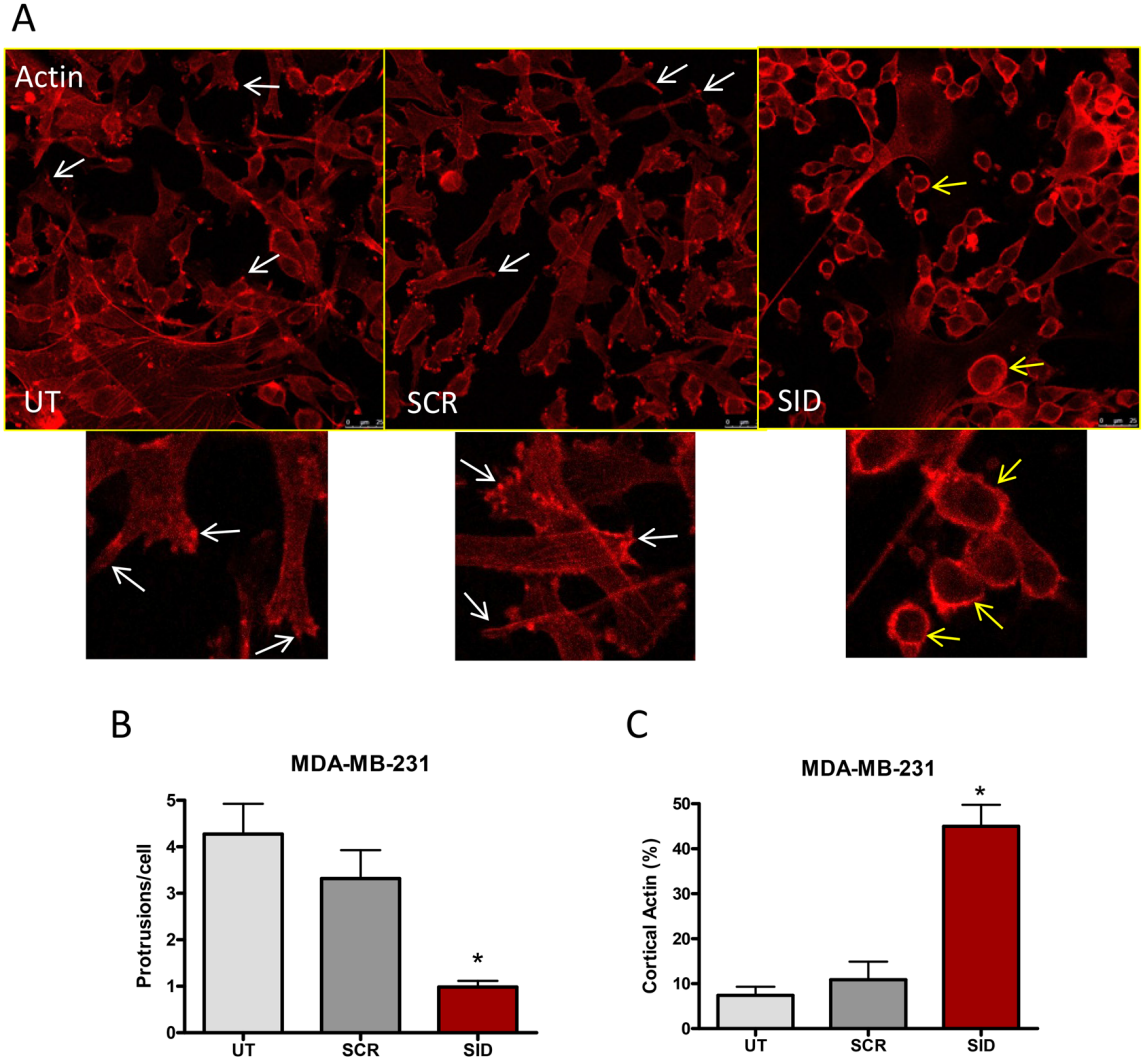
Induction of actin and microtubule cytoskeletal reorganization by SID peptide treatment in MDA-MB-231 cells **A.** MDA-MB-231 cells were treated with 2.5μM SID or SCR peptide for 24 hours and stained with rhodamine-phalloidin dye. MDA-MB-231 cells that were either treated with SCR peptide (SCR) or untreated (UT) show elongated cell shape with protruding edges (marked by white arrows), whereas MDA-MB-231 cells treated with SID peptide (SID) demonstrate cortical actin formation (yellow arrows). The images shown in Figure 1A are the representatives of multiple fields. **B.** The average number of protrusions per cell was quantified in UT, SCR, and SID treated cells. **C**. The percentages of cells with cortical actin in UT, SCR, and SID treated cells were calculated. (B; ^*^P<0.0004, C; ^*^P<0.0001, One-way ANOVA, error bars, mean ± SEM) (n of images =12 for UT, 10 for SCR, and 9 for SID, respectively).

Since cancer cell invasion and migration require dynamic cytoskeletal reorganization [20, 21], we next determined the influence of SID peptide on cell shape and organization of the actin cytoskeleton. MDA-MB-231 cells, cultured on a thin layer of Matrigel and treated with 2.5 μM SID peptide for 24 h, acquired a round and luminal-like appearance with loss of actin stress fibers network in comparison to the spindle-shaped mesenchymal-like cells in the untreated (UT) or SCR controls (Figure 2A). SID treatment resulted in 75% reduction in the number of actin-rich cell protrusions that generate the net force for cell movement [15] (Figure 2B, white arrows). Instead, the SID peptide treated cells displayed a strong cortical actin deposition in ∼45% of the cells in comparison to ∼10% in the UT or SCR-treated cells (Figure 2C, yellow arrows). Similar results were observed in the D3H2LN cells (Figure S2A). In these cells, SID peptide significantly reduced the number of actin protrusion per cell by ∼67% in comparison to UT or SCR-treated cells (Figure S2B). The number of SID-treated cells with a strong cortical actin deposition were ∼32% of the total population compared to ∼12% in the UT or SCR-treated cells (Figure S2C). These change in cytoskeleton dynamics are consistent with previously described effects of SID peptide on the reversion of the EMT and invasive phenotype [12].

### SID peptide down-regulates the Wnt and EMT pathways and induces nuclear export of β-catenin

**Figure 3:**
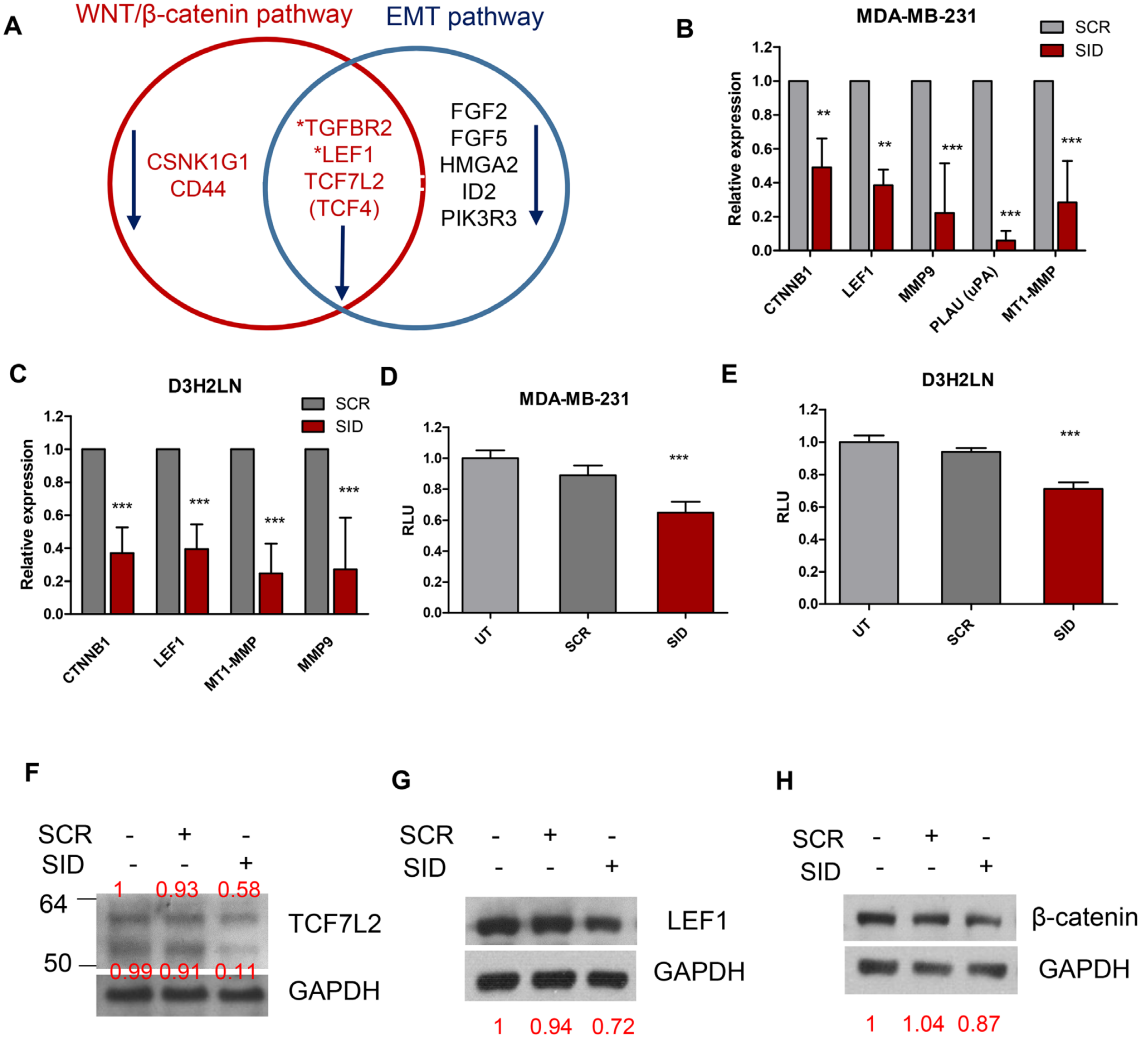
Treatment with SID decoy peptide leads to the inhibition of Wnt and EMT pathway genes **A.** The Venn diagram of gene expression microarray data shows that treatment with SID peptide (2.5μM, 24 hours) leads to downregulation of Wnt and EMT pathway genes by at least 2-fold in MDA-MB-231 cells. The detailed information is listed in Table 2 and 3. **B.** qPCR of CTNNB1, LEF1, MMP9, PLAU, MT1-MMP in MDA-MB-231 cells after SCR or SID treatment (2.5μM, 24 hours) (CTNNB1 and LEF1; ^**^<P<0.01, MMP9, PLAU, MT1-MMP; ^***^P<0.001, error bars, mean± SD, SID vs SCR, Two-way ANOVA) **C.** qPCR of CTNNB1, LEF1, MT1-MMP, MMP9 in D3H2LN cells as in (B). (^***^P<0.001, error bars, mean ± SD, SID vs SCR, Two-way ANOVA) **D, E.** Wnt reporter assay for MDA-MB-231 (D) or D3H2LN (E) cells. Cells were incubated with TOP flash and renilla lentiviral supernatant for 24 hours and treated with SCR or SID peptide (2.5μM) containing media for the next 24 hours. Wnt reporter activity was measured and calculated by normalizing the value of firefly to renilla luciferase activity. Relative luciferase unit (RLU) indicates Wnt reporter activity normalized to those of untreated cells (UT). (D; ^***^P=0.0006, E; ^***^P<0.0001, error bars, mean± SEM, SID vs. SCR or UT, One-way ANOVA) **F, G, H.** Western blot analysis of TCF7L2 (F), LEF1 (G), and β-catenin (H) protein in D3H2LN TNBC cells after treatment with SCR or SID decoy peptide (2.5μM, 24 hours). SID decoy treatment inhibited full-length (∼64 kDa) and, to a greater extent, short isoform variant (∼53 kDa) TCF7L2 proteins in comparison to untreated or SCR treated controls. The same treatment led to inhibition of LEF1 short isoform (∼40kDa), an active LEF1 protein. The band intensities for TCF7L2, LEF1, and β-catenin in each lane were normalized to the GAPDH loading control and indicated in red. The result is representative of three independent experiments.

Pathway analysis of expression microarray data obtained from the MDA-MB-231 cells treated with 2.5μM SID peptide for 24 hours (Supplementary File 1) identified WNT/β-catenin signaling as one of the top significantly altered canonical pathways (P-value= 4.64E-04) (Table 1 and 2 and Supplementary file 2-3). WNT/β-catenin signaling is one of the key pathways regulating EMT, cancer cell stemness, tumor invasion and metastasis [14, 17, 22]. We have previously reported that SID peptide inhibits the EMT pathway ([12] and Table 3) and Wnt target genes like CD44 (Figure 3A and Table 2) are downregulated by SID peptide. Other pathways modulated by SID peptide include Rho family GTPase signaling (P-value=1.90E-02) and Rho GDP signaling (P-value= 5.79E-04) (Table 1 and Supplementary File 2), which are involved in actin cytoskeletal reorganization and cell migration [23].

**Table 1:** Pathways differentially regulated by 2.5μM SID treatment (24hr)

Canonical pathway	P-value
Wnt/β-catenin Signaling	4.64E-04
Signaling by Rho Family GTPases	1.90E-02
RhoGDI Signaling	5.79E-04

**Table 2:** Genes involved in WNT/β-catenin pathway that are downregulated by 2.5μM SID treatment

Symbol	Entrez Gene Name	Log Ratio(24h)
CD44	CD44 molecule (Indian blood group)	-1.600
CSNK1G1	casein kinase 1, gamma 1	-1.030
LEF1	lymphoid enhancer-binding factor 1	-1.110
TCF7L2	transcription factor 7-like 2 (T-cell specific, HMG-box)	-1.060
TGFBR2	transforming growth factor, beta receptor II (70/80kDa)	-1.330

**Table 3:** Genes involved in the regulation of the EMT pathway that are downregulated by 2.5μM SID treatment

Symbol	Entrez Gene Name	Log Ratio(24h)
FGF2	fibroblast growth factor 2 (basic)	-1.480
FGF5	fibroblast growth factor 5	-1.260
HMGA2	--	-1.240
ID2	inhibitor of DNA binding 2, dominant negative helix-loop-helix protein	-1.390
LEF1	lymphoid enhancer-binding factor 1	-1.110
LOX	lysyl oxidase	1.230
PIK3R3	phosphoinositide-3-kinase, regulatory subunit 3 (gamma)	-1.290
TCF7L2	transcription factor 7-like 2 (T-cell specific, HMG-box)	-1.060
TGFBR2	transforming growth factor, beta receptor II (70/80kDa)	-1.330

This suggested inhibition of Wnt pathway was confirmed by measuring the expression of additional target Wnt target genes (*CTNNB1, LEF1, MMP9, MT1-MMP, PLAU)* in MDA-MB-231 cells treated with SID peptide. Treatment with SID peptide significantly decreased the expression of all the genes tested by qRT-PCR (CTNNB1, 2-fold; LEF1, 2.6-fold; MMP9, 4.5- fold; PLAU, 16.7-fold; MT1-MMP/MMP14, 3.6- fold) (Figure 3B). Downregulation of these genes upon SID peptide treatment was also confirmed in D3H2LN cells (CTNNB1, 2.7-fold; LEF1, 2.5-fold; MT1-MMP/MMP14, 4-fold; MMP9, 3.7-fold) (Figure 3C). Functional inhibition of Wnt/β-catenin signaling by SID decoy peptide was confirmed by measuring the WNT/β-catenin reporter activity, using the promoter that contains multiple consensus TCF binding sites also called Wnt responsive element (WRE) (5’-CCTTTGWW-3’, W=A or T) [24]. SID peptide treatment led to modest but statistically significant inhibition of WNT luciferase activity by 35% in MDA-MB-231 and 29% in D3H2LN cells compared to untreated and SCR treated cells (Figure 3D-3E). Western blot analysis of protein level and immunofluorescence staining for three target genes, β-catenin, LEF1 and TCF7L2 showed that SID peptide treatment resulted in decrease of TCF7L2 and LEF1 (Figure 3F-3G, S4A-S4B). There was some inhibition of the total β-catenin level by SID decoy but it was not significantly affected by the treatment in D3H2LN cells (Figure 3H).

**Figure 4:**
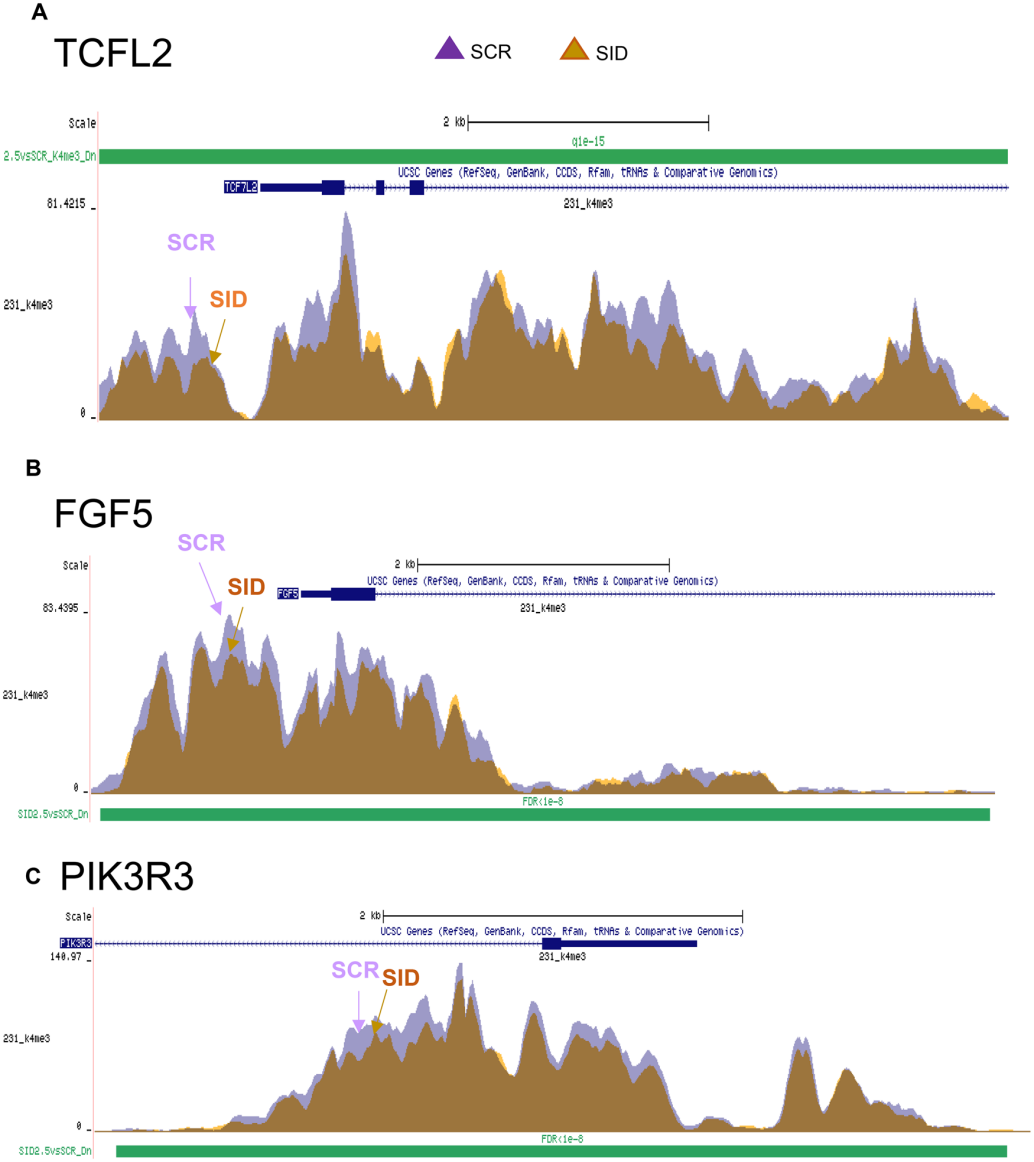
SID peptide treatment markedly inhibits H3K4Me3 marks in a set of Wnt and EMT pathway genes **A.** ChIP-seq analysis for the H3K4Me3 mark bound to TCF7L2 gene. SID peptide treatment decreases histone H3K4Me3 peak level in the proximal gene promoter. Purple; SCR, orange; 2.5μM SID (FDR<1x10^-15^). **B, C.** ChIP-seq analysis for the H3K4Me3 mark bound to FGF5 (B) and PIK3R3 (C) genes (FDR<1X10^-8^).

We have previously reported that SID peptide treatment induces global epigenetic changes marked by overall reduction of the active transcription mark histone 3 lysine 4 tri-methylation (H3K4Me3) occupancy in a cluster of gene sets [12]. We examined if Wnt and EMT pathway genes downregulated after SID peptide treatment (Figure 4A-4C, Table 2, 3) were also marked by reduction in H3K4Me3. ChIP-seq data [12] were compared with DNA microarray results (Supplementary File 1). Interestingly, TCF7L2 (Figure 4A, Table 2, 3) and CD44 [12] genes are in the list of Wnt pathway genes in which H3K4Me3 bound to the gene promoters was significantly decreased after SID decoy treatment (2.5 μM, 24 hours) (FDR<1X10^-15^) (Figure 4A, [12]). EMT pathway genes (e.g., FGF5 and PIK3R3) downregulated by SID decoys in the microarray also showed reduced level of histone H3K4Me3 bound to the genes (FDR<1X10-^8^) (Figure 4B-4C). Taken together, SID peptide regulates H3K4Me3 epigenetic marks in a cluster of WNT and EMT pathway promoting genes.

**Figure 5:**
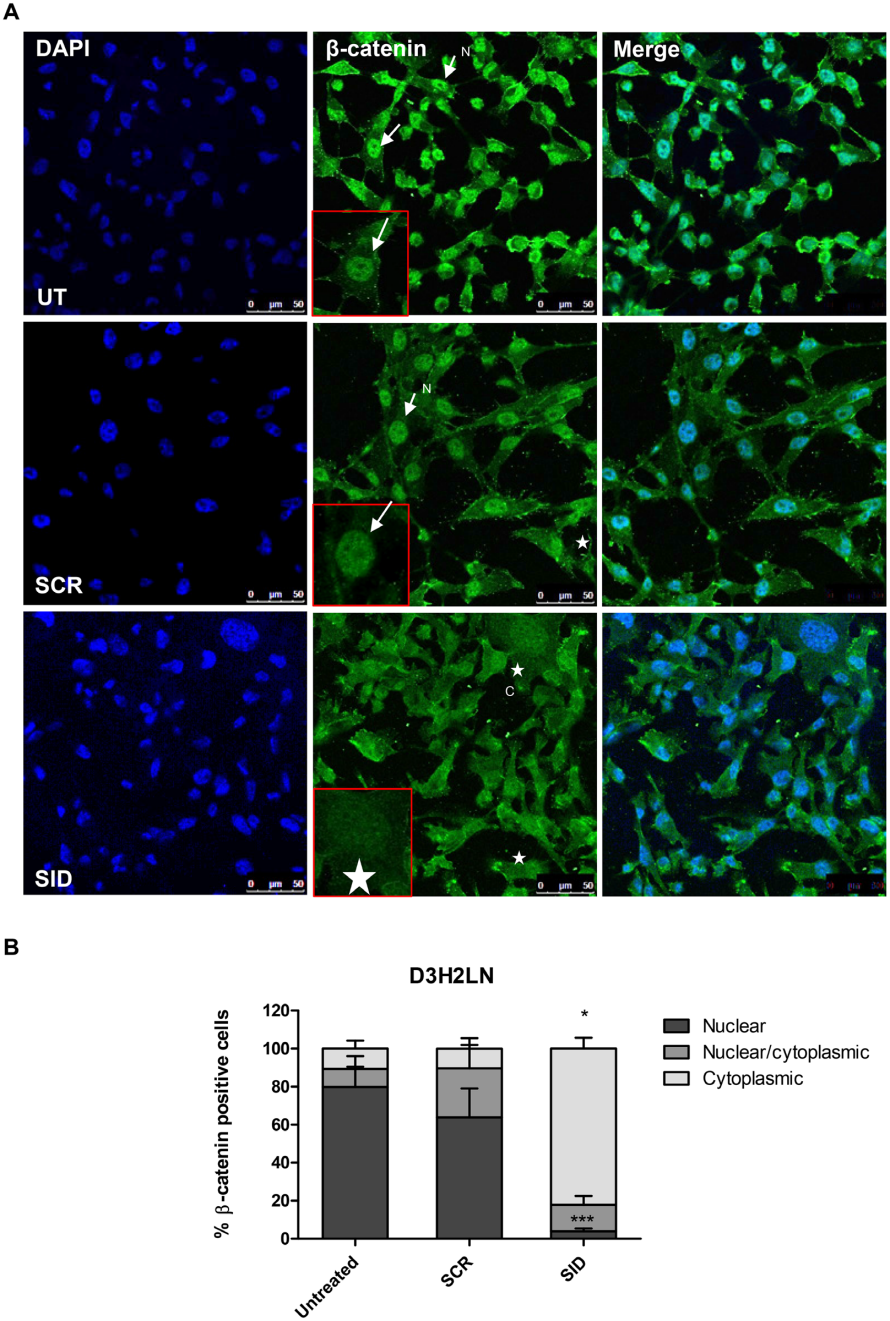
Treatment with SID decoy peptide induces translocation of nuclear β-catenin to the cytoplasmic compartment in TNBC cells **A.** D3H2LN cells were either untreated or treated with SCR or SID peptide (2.5μM) for 24 hours and stained with β-catenin mAb. In untreated (UT) or SCR treated D3H2LN cells, β-catenin is localized mostly in the nucleus (arrows). In D3H2LN cells treated with SID decoy peptide, β-catenin is distributed into the cytoplasm (stars). N-nuclear; C- cytoplasmic **B.** Quantification of % cells with nuclear, nuclear/cytoplasmic, and cytoplasmic β-catenin localization in untreated, SCR treated, or SID treated D3H2LN cells. The graph represents a compilation of the results from three experiments. (Nuclear; ^***^P=0.0002, cytoplasmic,^*^P<0.0001, SID vs. SCR or untreated, One-way ANOVA, error bar, mean ± SEM).

Since only nuclear β-catenin co-opts with Wnt signaling [24], we also examined the effects of SID peptide on cellular localization of β-catenin. In comparison to SCR peptide, SID peptide treatment (2.5μM, 24 hours) induced relocation of β-catenin into the cytoplasm in both D3H2LN and MDA-MB-157 cells. In D3H2LN cells, following SID treatment only 4% cells retained nuclear β-catenin while in SCR treated cells, nuclear β-catenin was observed in 64% of the total population (Figure 5A-5B). Similarly in MDA-MB-157 cells 60% of SCR treated cells had nuclear β-catenin compared to only 30% in SID treated cells (Figure S3A-S3B). Membrane-associated β-catenin was not observed in SID treated cells as found in our previous study [10]. However, in that study, SID peptide was expressed from stably transfected vector over a longer time period. Taken together, these results indicate that SID peptide inhibits the Wnt/β-catenin pathway that may contribute to impairment of the early stages of invasion of TNBC cells.

### Disruption of the interaction of PAH2 domain of SIN3A with TGIF1 markedly inhibits invasion and Wnt/β-catenin signaling

**Figure 6:**
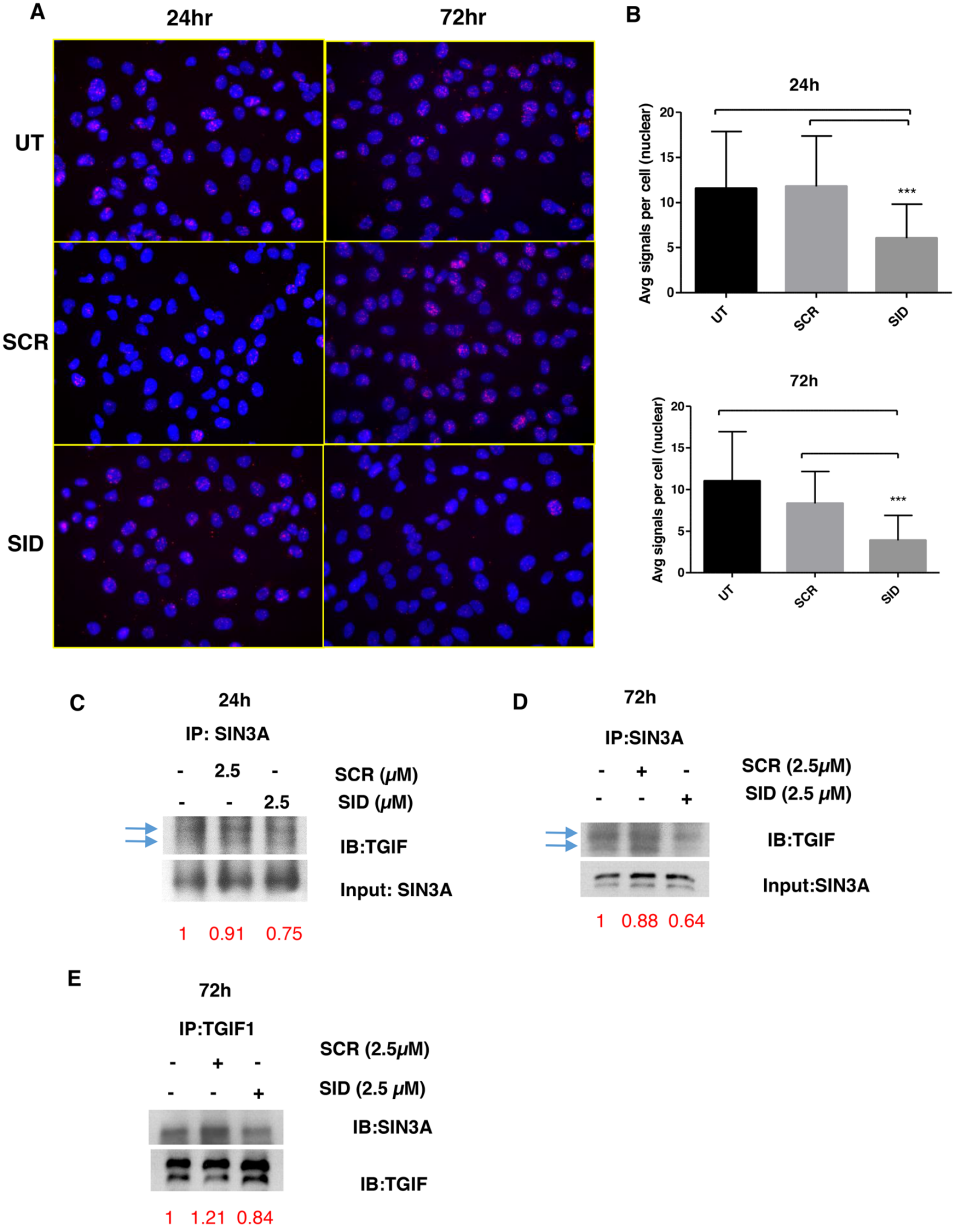
SID decoy peptide interferes with the interaction between SIN3A and TGIF1 protein **A.** Proximity Ligation Assay (PLA) was performed for MDA-MB-231 cells either untreated or treated with SCR or SID peptide (2.5μM, 24 or 72 hours). **B.** The average number of signals per cell in the nucleus was quantified from three images per sample from the same experiments as in (A). (^***^P<0.001, SID vs. SCR or untreated (UT), One-way ANOVA, error bar, mean± SEM) **C, D.** Nuclear extract of SCR or SID treated (2.5μM, 24 (C) or 72 hours (D)) or UT cells was used for immunoprecipitation of SIN3A protein using SIN3A polyclonal antibody (sc 767) and immunoblotted with TGIF1 antibody. The band intensities for TGIF1 immunoblotting (IB) (C, D) were normalized to those of the SIN3A input loading and indicated in red. **E.** Nuclear extract of SCR or SID treated (2.5μM, 72 hours) or UT cells were used for immunoprecipitation of TGIF1 protein using TGIF1 polyclonal antibody (H-172) and immunoblotted with SIN3A (E) antibody. The band intensities for SIN3A (E) IB were normalized to those of TGIF1 IB, which was indicated in red.

We next questioned if the inhibition of invasion and Wnt signaling by SID peptide is due to disruption of protein interactions specific to the PAH2 domain of SIN3A. Of the limited number of proteins known to interact with PAH2 domain [7], the SID-containing transcription factor TGIF1 [25], is known to modulate Wnt signaling [26]. We first determined if TGIF1 was displaced from the SIN3A complex by SID peptide. Proximity ligation assay (PLA) was performed in MDA-MB-231 cells that were treated with 2.5μM SID peptide for 24 and 72 hours. In comparison to the SCR peptide, SID peptide treatment led to significant 48 % and 65% decrease in SIN3A and TGIF1 interaction after 24 h and 72 h (Figure 6A-6B) without overt changes in the level and the localization of TGIF1 protein (Figure S5). These results were further confirmed by SIN3A co-immunoprecipitation (co-IP) with TGIF1 antibody in the nuclear fraction in which the SIN3A-TGIF1 binding was reduced (by 25% and 36% after 24 and 72 hours of treatment, respectively) in D3H2LN cells treated with 2.5μM SID peptide (Figure 6C-6E).

**Figure 7:**
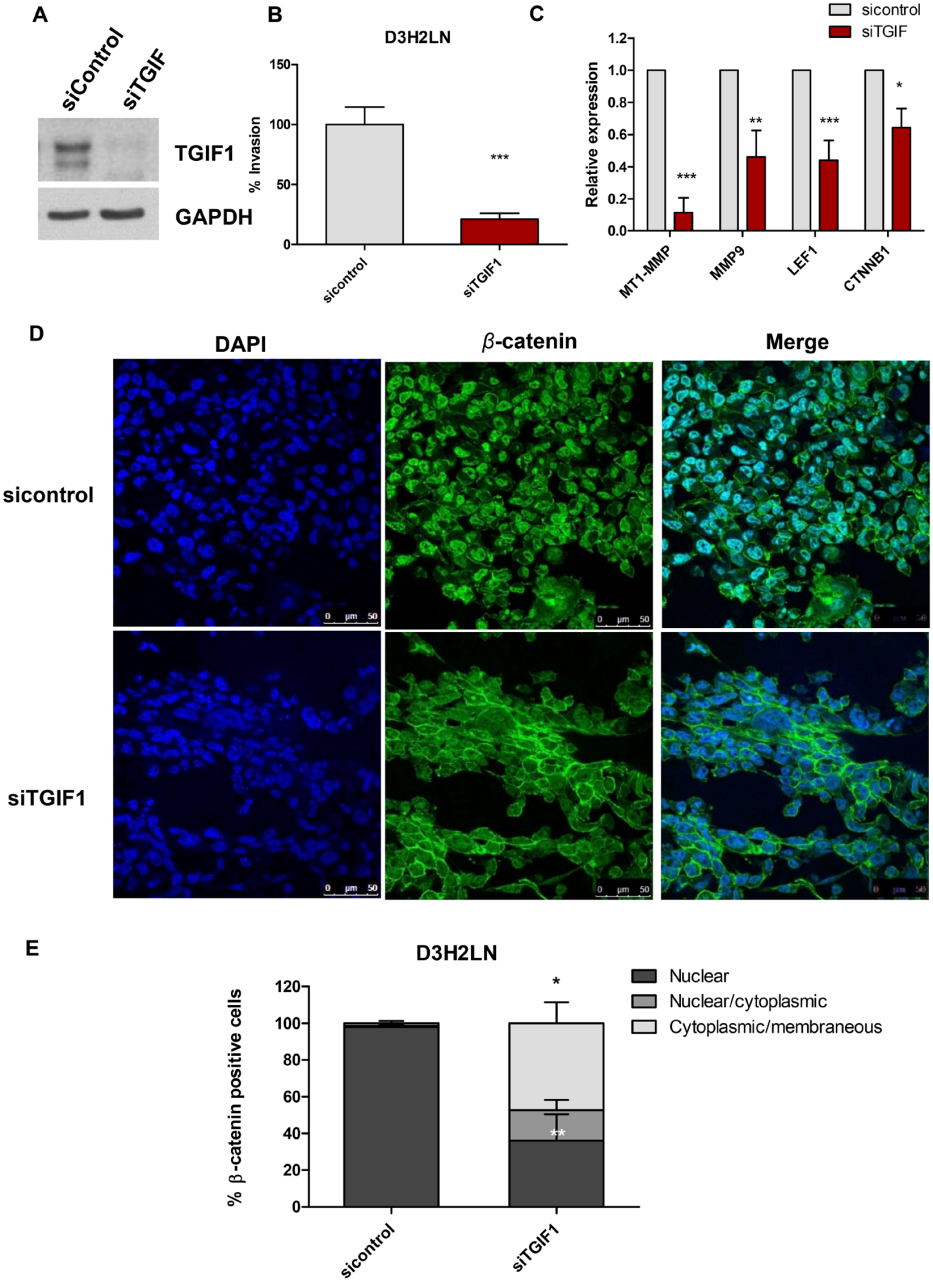
Knockdown of TGIF1 in D3H2LN cells suppresses transwell invasion and Wnt/β-catenin target genes **A.** D3H2LN cells were transfected with 40nM siTGIF and immunoblotted with TGIF1 antibody at 48 hour post-transfection **B.** D3H2LN cells transfected with siTGIF were seeded on the upper wells of the inserts for transwell invasion assays at 48 hour post-transfection. The transwell invasion assays were performed. (^*^P<0.0001, siTGIF vs siControl, unpaired t test, mean±SEM) **C.** qPCR analysis for Wnt pathway target genes (MT1-MMP, MMP9, LEF1, CTNNB1) in D3H2LN cells transfected with siTGIF and siControl (MT1-MMP, LEF1; ^***^P<0.001, MMP9; ^**^P<0.01, CTNNB1; ^*^P<0.05, Two-way ANOVA, error bar, mean± SEM). **D.** Immunofluorescence staining of β-catenin in D3H2LN cells transfected with siControl (top) and siTGIF (bottom). **E.** The percentage of β-catenin positive cells was quantified from D3H2LN cells transfected with siControl and siTGIF1 for 48 hours (error bars, mean ± SEM, ^***^P<0.001, One way ANOVA followed by Tukey’s multiple comparison test, ^**^P<0.05; nuclear β-catenin in siTGIF vs. siControl, ^*^ P<0.05; cytoplasmic/membranous β-catenin in siTGIF vs. siControl).

To further investigate the role of SIN3A-TGIF1 interaction in invasion and Wnt signaling, TGIF1 protein was depleted in D3H2LN cells by transfecting a pool of three TGIF1-targeting siRNAs (siTGIF1) (Figure 7A). A marked 80% reduction of cell invasion was observed in cells transfected with siTGIF1 (Figure 7B). No changes in cell growth were noted in siTGIF1-transfected cells (data not shown). TGIF1 knockdown also resulted in significant inhibition of Wnt target genes, including MT1-MMP, MMP-9 mRNA levels by 10 and 2-fold, respectively at 48 hour post-transfection (Figure 7C). Other Wnt target genes, such as LEF1 and CTNNB1 were also downregulated by up to 2-fold in D3H2LN cells transfected with siTGIF1 (Figure 7C). Similar to SID peptide, knocking down TGIF1 resulted in nuclear export of β-catenin with a strong membrane associated β-catenin as previously reported with stable transfections of SID peptide [10] (Figure 7D-7E). Taken together, these data clearly demonstrate that TGIF1 knockdown phenocopies SID peptide treatments and the resulting inhibition of invasion and Wnt/β-catenin signaling may be linked to disruption of SIN3A-TGIF1 interaction by SID peptide.

## DISCUSSION

Therapeutic intervention for TNBC is hampered due to tumor cell heterogeneity and lack of major molecular targets [4]. Recent advances in the molecular profiling from *in vitro* cultured tumor cell lines and patient samples led us to identify novel genetic and epigenetic aberrations that contribute to tumor progression, which could be utilized for the development of novel targeted therapies [27, 28]. Previously we reported that SID decoy peptide designed to selectively target the PAH2 domain of SIN3 complex induced epigenetic reprogramming in TNBC [10]. We reported that SID decoy peptide and small molecule inhibitor (SMI) mimetic exert anti-tumor and anti-metastatic activity *in vivo* in TNBC xenograft mouse models with minimum cytotoxicity [10, 11]. In TNBC, the effect of SID peptide or SMI mimetic did not involve growth inhibition in 2-dimensional (2D) culture condition but significant inhibition of invasive colony outgrowth in 3D cultures in Matrigel, with partial acini differentiation, as well as inhibition of tumor spheroid formation in suspension culture, representing cancer stem-like cells [10–12].

Here, we show that by blocking the interaction of SIN3A with selective partners (transcription factors and chromatin modifiers) through its PAH2 domain using SID peptide, the invasive phenotype of TNBC cells is suppressed and that this process is mediated by the impairment of Wnt signaling. We anticipated that SID peptide might also modulate Wnt pathway since ivermectin, shown previously to have a SID decoy mimetic activity [11], also suppresses Wnt pathway in colorectal cancer [29]. Our data show that SID peptide downregulates Wnt/β-catenin reporter activity and direct Wnt target genes (LEF1, TCF7L2, CD44, PLAU, MT1-MMP, MMP9, HMGA2) involved in breast cancer invasion and metastasis (Figure 3A-3E, Table 2, 3). The involvement of Wnt signaling pathway, including LEF1 and TCF7L2, in the promotion of invasion, CSC self-renewal, and metastasis of TNBC as well as other tumor types (i.e., lung and prostate) had been established [15, 18, 30–37]. In particular, TCF7L2 was shown to promote breast cancer cell invasion by upregulation of miR-21 [38] and Wnt ligand induced β-catenin activation is also known to enhance invasion and metastasis of TNBC cells [22, 39], suggesting the regulation of invasion could be mediated by modulating Wnt pathway in TNBC.

Our results show that SID peptide attenuates Wnt/β-catenin pathway activity, at least in part, by promoting the translocation of nuclear β-catenin into the cytoplasm (Figure 5, Figure S3) that leads to the reduced Wnt reporter activity and the expression of Wnt target genes (Figure 3A-3E, Table 2). Since TGIF1 is a positive regulator for Wnt signaling and also a SIN3A PAH2 domain interacting protein [25, 26], we investigated whether TGIF1 plays a role in SID decoy mediated inhibition of invasion and found that this could be mediated through inhibition of Wnt pathway. We suggest that the moderate level of disengagement of TGIF1 from SIN3 complex upon SID peptide treatment (Figure 6) could result in attenuation of TGIF1 function. Our data indicate that cells treated with SID peptide phenocopies the effect of TGIF1 knockdown, such as delocalization of β-catenin from the nuclei (Figure 5A-5B) and inhibition of some Wnt pathway target genes (Figure 3A-3C). The alteration of invasive potential through modulation of TGIF1 level is well supported by the recent publications in which TGIF1 enhances proliferation, migration, invasion, and colony outgrowth in cancerous and noncancerous cells of different origins [40–42]. Consistent with our findings, TGIF1 is overexpressed in TNBC as compared to other breast cancer subtypes and is associated with hyperactive Wnt signaling marked by increased β-catenin accumulation and its target expression [26]. Increased TGIF1 level correlates with a poorer outcome and a higher disease relapse in TNBC patients [26], suggesting that TGIF1 could be identified as a potential biomarker and a druggable target for anti-TNBC therapy.

Our data and others indicate that the SIN3A or Sin3B/HDAC complex(es), generally known as a part of transcriptional repressor complexes [25], can mediate gene activation by regulating the levels of H3K4Me3 marks bound to actively transcribing genes [43–46]. We have shown that decreased H3K4Me3 occupancy on EMT and Wnt pathway genes was observed after SID decoy treatment (Figure 4A-4C), which correlates with the downregulation of EMT and Wnt pathway genes (Figure 3A, Table 2-3). Many of those genes inhibited by SID peptide treatment (CD44, TGFβR2, LEF1, TCF7L2, FGF2, FGF5, ID2, PIK3R3) (Figure 3A, Table 2-3) are known as direct TGIF1 targets in myeloid leukemia [47] or embryonic stem cells [48]. This suggests that SID decoys could inhibit transcription of TGIF1 target genes by interference with SIN3A-TGIF1 interaction in TNBC cells.

Treatment with SID peptide for 24 hours downregulates Wnt pathway targets and EMT-related genes and suppresses invasion in TNBC cells. We found that SIN3A in complex with TGIF1 promotes TNBC invasion and Wnt/β-catenin pathway, which is inhibited by SID decoys by attenuating SIN3A-TGIF1 interaction. E-cadherin is induced 72 hours after SID peptide treatment in TNBC [12], which is one of the later events leading toward luminal differentiation and reversion of EMT as compared to the inhibition of Wnt pathway activity and invasion in TNBCs. Therefore, induced E-cadherin in TNBC after SID peptide treatment could mediate the late events (∼after 24 hours) involved in the reversion of EMT and inhibition of invasion following Wnt pathway inhibition. Consistent with the concept, we observed that SID decoy treatment as well as TGIF1 knockdown leads to the downregulation of Wnt reporter activity with changes in β-catenin sub-cellular localization from nucleus to the cytoplasm, suggesting the overlap between SID decoy treatment and TGIF1 knockdown. These results suggest the reversion of EMT and invasion processes with SID decoy treatment has begun at least as early as 24 hours.

We have shown that SID decoys could be a novel anti-metastatic therapy for TNBC by selectively targeting TGIF1, one of the SID containing transcription factors. Targeted inhibition of TGIF1 by SID decoys leads to the inhibition of hyperactive Wnt pathway which occurs in about 40-60 % of TNBC cases [13, 14, 17]. SID peptides and small molecule inhibitors (SMIs) demonstrate substantial inhibition of tumor growth and metastases *in vivo* with minimum cytotoxicity [10–12], which would make it beneficial to use SID decoys as an adjuvant therapy in combination with existing chemotherapeutic agents. Currently, studies are underway to test efficacy of SID decoy SMIs in pre-clinical models *in vivo* to explore their potential use in TNBC patients.

## MATERIALS AND METHODS

### Cell culture and media

The human MDA-MB-231 breast adenocarcinoma (Cat# HTB-26) and MDA-MB-157 breast medullary carcinoma (Cat# HTB-24) cell lines were purchased from the American Type Culture Collection (ATCC). The MDA-MB-231-Luc-D3H2LN Bioware (D3H2LN) cell line was purchased from PerkinElmer (Cat# 119369). Mouse 4T1 cells (Cat# CRL-2539) were maintained in RPMI supplemented with 10% fetal bovine serum (FBS) and 1% Antibiotic-Antimycotic solution (Invitrogen). D3H2LN, MDA-MB-231, and MDA-MB-157 cell lines were maintained in DMEM supplemented with 10% FBS, 1% GlutaMAX (Invitrogen), 10mM HEPES, 1mM sodium pyruvate, non-essential amino acids and 1% antibiotic-antimycotic solution.

### Generation of SID decoy peptide

SID and scramble (SCR) peptides were generated as previously described [10, 12]. SID peptide constitutes TAT (underlined) sequences followed by N-terminal SID region of MAD1 (MXD1) protein (YGRKKRRQGGG-VRMNIQMLLEAADYLERRER). SCR peptide in addition to TAT peptide has the following sequence: YGRKKRRQGGG-EQRARRIMERLLEYNMVADL.

### Western blot analysis and immunoprecipitation

Immunoblotting was performed according to the standard procedures and visualized using ECL plus Western Blotting detection reagent (Life Technologies). Blots were probed with anti-β-catenin rabbit mAb (Cell Signaling Technology, catalog #8480), SIN3A (AK-11) antibody (Santa Cruz Biotechnology, catalog #sc-767), TGIF1 antibody (Santa Cruz Biotechnology, H-172), and anti-GAPDH antibody (Ambion, #4300). Immunoprecipitation was performed according to the standard procedures.

### Transwell invasion and migration assays

TNBC cells (5x10^4^) were seeded onto Matrigel-coated a 24-well filter insert (Corning) and 5% FBS was used as chemoattractant in the lower chamber. The numbers of invading cells were counted after 24 hours, stained with HEMA 3 stain set (Fisher Scientific), and the number of invasive cells after treatment was normalized to that of untreated cells (UT) and calculated as the percentage of invasive cells. The total 5 fields were counted per filter under 200x magnification using a Nikon Eclipse E600 microscope. For transwell migration assay, cells were seeded onto the top well of a 24-well filter insert without Matrigel coating and the same procedures as invasion assays were performed afterwards.

### Immunofluorescence

D3H2LN or MDA-MB-157 cells were plated onto 8 well chamber coated with thin layer of Matrigel diluted in PBS (1:100 dilution) and treated with SCR or SID peptide (2.5μM) for 24 hours. Cells were stained with rabbit β-catenin (D10A8) mAb (Cell signaling technology, cat#-8480), TCF7L2 (Novus Biologicals, cat# NBP-19083), rabbit LEF1 (C12A5) (Cell signaling technology, cat#2230P) or rabbit polyclonal TGIF1 (H-172) antibody (Santa Cruz Biotechnology, sc-9084) 1:100-1:250 overnight at 4°C according to standard protocols. Cells were fixed with 4% paraformaldehyde at RT for 15 min and incubated with 0.5% Triton-X-100 at 37°C for 5 min, and blocked with 5% bovine serum albumin at RT for 1 hour. Secondary antibodies (dilution 1:200 in 1% normal goat serum/PBS) were added for 1 h and then washed with PBS. Cells were mounted in Prolong Gold antifade reagent (Life technologies, ref # P36931) Images were collected on a Leica SP5 confocal microscope.

### Quantitation of β-catenin nuclear and cytoplasmic localization

The number of cells with “nuclear”, “nuclear/cytoplasmic”, or “cytoplasmic” β-catenin was quantified from immunofluorescence images (6-9 fields quantified per sample). For quantification, we used plugins -> particle analysis-> cell counter menu in ImageJ program (NIH). Cells that have fluorescence intensity above designated threshold level in ImageJ were considered as β-catenin positive cells. The number of “nuclear”, “nuclear/cytoplasmic”, or “cytoplasmic” β-catenin positive cells was normalized to the total number of cells counted from multiple fields and converted into the percentage of β-catenin positive cells. The cells with “nuclear β-catenin” staining demonstrate those with β-catenin localization exclusively in the nucleus. The cells with “nuclear/cytoplasmic β-catenin” show distribution of nuclear localization of β-catenin that are weaker than cells with “nuclear β-catenin” and have some cytoplasmic distribution. The cells with “cytoplasmic β-catenin” show ubiquitous localization of β-catenin staining without strong fluorescence signal concentrated in either nucleus or cytoplasm.

### WNT/β-catenin luciferase activity

NV-Ins Top Luc (Top flash) and NV-Ins Fop Luc (Fop flash) constructs, and renilla supernatant, were generously provided by Dr. Stuart Aaronson lab in Mount Sinai. To generate lentiviral supernatant, 293T cells were co-transfected with 5μg of TOP flash construct with packaging plasmids (3.75μg of NRF, 1.75μg of VSVG) when cells reached 80% confluency. Supernatant was harvested and used for transduction of lentiviral particles into MDA-MB-231 and D3H2LN cells, respectively. For transduction, 2X10^4^ cells were plated per well on 24-well plates and transduced with Top flash and Fop flash supernatant. The next day, cells were treated with 2.5μM SID or SCR peptide in regular media containing 5% Fetal Bovine Serum (FBS) for 24 hours and reporter assays were performed using the Dual Luciferase reporter assay system (Promega) according to the manufacturer’s instructions. Firefly luciferase activity was normalized to renilla luciferase activity and demonstrated as Wnt reporter activity.

### Quantitative RT-PCR (qRT-PCR)

RNA was prepared using RNeasy Plus Mini kit (Qiagen). For real-time qPCR, 1μg RNA was reverse-transcribed with iScript cDNA Synthesis kit (Bio-Rad). 50-250 ng of resulting cDNA were amplified and analyzed in real time with iTaq™ Universal SYBR^®^ Green Supermix (Bio-rad). Results are represented after values were normalized to housekeeping genes (*RPL30* or *GAPDH*) and are presented as fold-differences over control using the ∆∆Ct method for relative quantifications. Each comparison was made using triplicate reactions and in at least 3 experiments. Primer sequences are described in details in Table S1.

### Transfection of siRNA

Cells were plated 40-50 percent confluent in 10 cm plates on the day of transfection. Silencer select non-targeting negative control siRNA (Ambion) or a pool of 4 different siRNAs targeting TGIF1 (40nM) (Dharmacon siGENOME, GE lifesciences) were used for transfection. Cells were used for invasion assays and qPCR analysis at 48 hour post-transfection.

### Affymatrix gene microarray and ingenuity pathway Analysis (IPA)

Affymatrix gene microarray was performed in MDA-MB-231 cells that were either untreated or treated with 2.5μM SCR or SID peptide for 24 hours as previously described [12]. Total RNA was isolated using the ZR RNA MiniPrep Kit (Zymo Research). The concentration and quality of the total RNA was assessed on an Agilent 2100 BioAnalyzer (Agilent Technologies). All samples were normalized to 200ng and processed according to standard Affymetrix protocols using GeneChip WT Terminal Labeling and Controls Kit (Affymetrix) and WT Expression Kit (Ambion). The quality and quantity of labeled cRNA was checked and 750 ng of labeled cRNA was hybridized to a GeneChip Human Gene 1.0 ST Arrays using GeneChip Hybridization, Wash, and Stain Kit (Affymetrix). The arrays were scanned on a GeneChip Scanner 3000 7G. Affymetrix array data were analyzed by Chipinspector 2.1 (Genomatix). Transcripts were considered significantly regulated if at least 3 significant probes mapped to them and the log2 fold change of the transcript calculated from these probes was above 1 or below -1. For all subsequent analyses, we used the median expression values of two independent biological replicates. Replicates were combined exhaustively, i.e. mean fold changes were calculated by comparing each replicate from the treatment group to each replicate from the control group. Log2 fold change values for genes were calculated as the average of the log2 fold change values of the corresponding significantly regulated transcripts and a False Discovery Rate (FDR) was set as 5%. Expression microarray analysis was performed according to Minimum Information About a Microarray Gene Experiment (MIAME) guidelines and data have deposited on the Gene Expression Ontology (GEO) database with the series accession number GSE84648. To identify significantly altered genes and signaling pathways in MDA-MB-231 cells after treatment with SID peptide (2.5μM, 24 hours) in comparison to 2.5μM SCR peptide treatment, the gene microarray data were uploaded and analyzed using IPA software. At least 2-fold gene expression changes (less than -1 or more than +1 of the log2 fold changes) were used as a cut-off for the significantly regulated genes after SID peptide treatment.

### Chromatin immunoprecipitation (ChIP)-seq analysis

The native ChIP-seq data for H3K4Me3 that had been previously published [12] were used to observe the H3K4Me3 occupancy in the promoters of Wnt/β-catenin and EMT pathway genes. The ChIP data are available on the Gene Expression Ontology (GEO) database with accession number GSE 73869. Briefly, MDA-MB-231 cells that were either untreated or treated with 2.5μM SCR or SID peptide for 24 hours were used for ChIP-seq experiment previously published [12]. To find significantly changed peaks between untreated and SID treated MDA-MB-231 cells, the following parameters were used in SICER-df program [49]. For H3K4Me3: window size = 50bp, gap size = 400bp, island calling = FDR<1x10^-4^, SCR versus SID = FDR<1x10^-8^. We determined Wnt and EMT target genes with significant changes in the H3K4Me3 occupancy by intersecting significantly changed peaks of H3K4Me3 ChIP to ±3 Kb TSS of all Refseq genes, respectively, using Bedtools. Histone modification snapshots were generated using UCSC Genome Browser.

### Statistical analysis

Statistical analyses were performed using a one-way analysis of variance (ANOVA) to compare all pairs of experimental groups. Data were represented as mean ± SEM (or SD) for replicate values. P <0.05 was regarded as statistically significant. For comparison of two groups, two-tailed student’s t tests were used. All the statistical analyses were performed using Graphpad Prism software.

## SUPPLEMENTARY MATERIALS FIGURES, TABLE AND FILES









## References

[1] Foulkes WD, Stefansson IM, Chappuis PO, Begin LR, Goffin JR, Wong N, Trudel M, Akslen LA (2003). Germline BRCA1 mutations and a basal epithelial phenotype in breast cancer. J Natl Cancer Inst.

[2] Lakhani SR, Van De Vijver MJ, Jacquemier J, Anderson TJ, Osin PP, McGuffog L, Easton DF (2002). The pathology of familial breast cancer: predictive value of immunohistochemical markers estrogen receptor, progesterone receptor, HER-2, and p53 in patients with mutations in BRCA1 and BRCA2. J Clin Oncol.

[3] Carey LA, Perou CM, Livasy CA, Dressler LG, Cowan D, Conway K, Karaca G, Troester MA, Tse CK, Edmiston S, Deming SL, Geradts J, Cheang MC (2006). Race, breast cancer subtypes, and survival in the Carolina Breast Cancer Study. JAMA.

[4] Turner NC, Reis-Filho JS (2013). Tackling the diversity of triple-negative breast cancer. Clin Cancer Res.

[5] Foulkes WD, Smith IE, Reis-Filho JS (2010). Triple-negative breast cancer. N Engl J Med.

[6] Dent R, Trudeau M, Pritchard KI, Hanna WM, Kahn HK, Sawka CA, Lickley LA, Rawlinson E, Sun P, Narod SA (2007). Triple-negative breast cancer: clinical features and patterns of recurrence. Clin Cancer Res.

[7] Bansal N, David G, Farias E, Waxman S (2016). Emerging Roles of Epigenetic Regulator Sin3 in Cancer. Adv Cancer Res.

[8] Ellison-Zelski SJ, Solodin NM, Alarid ET (2009). Repression of ESR1 through actions of estrogen receptor alpha and Sin3A at the proximal promoter. Mol Cell Biol.

[9] Ellison-Zelski SJ, Alarid ET (2010). Maximum growth and survival of estrogen receptor-alpha positive breast cancer cells requires the Sin3A transcriptional repressor. Mol Cancer.

[10] Farias EF, Petrie K, Leibovitch B, Murtagh J, Chornet MB, Schenk T, Zelent A, Waxman S (2010). Interference with Sin3 function induces epigenetic reprogramming and differentiation in breast cancer cells. Proc Natl Acad Sci U S A.

[11] Kwon YJ, Petrie K, Leibovitch BA, Zeng L, Mezei M, Howell L, Gil V, Christova R, Bansal N, Yang S, Sharma R, Ariztia EV, Frankum J (2015). Selective Inhibition of SIN3 Corepressor with Avermectins as a Novel Therapeutic Strategy in Triple-Negative Breast Cancer. Mol Cancer Ther.

[12] Bansal N, Petrie K, Christova R, Chung CY, Leibovitch BA, Howell L, Gil V, Sbirkov Y, Lee E, Wexler J, Ariztia EV, Sharma R, Zhu J (2015). Targeting the SIN3A-PF1 interaction inhibits epithelial to mesenchymal transition and maintenance of a stem cell phenotype in triple negative breast cancer. Oncotarget.

[13] Khramtsov AI, Khramtsova GF, Tretiakova M, Huo D, Olopade OI, Goss KH (2010). Wnt/beta-catenin pathway activation is enriched in basal-like breast cancers and predicts poor outcome. Am J Pathol.

[14] Geyer FC, Lacroix-Triki M, Savage K, Arnedos M, Lambros MB, MacKay A, Natrajan R, Reis-Filho JS (2011). beta-Catenin pathway activation in breast cancer is associated with triple-negative phenotype but not with CTNNB1 mutation. Mod Pathol.

[15] Dey N, Barwick BG, Moreno CS, Ordanic-Kodani M, Chen Z, Oprea-Ilies G, Tang W, Catzavelos C, Kerstann KF, Sledge GW, Abramovitz M, Bouzyk M, De P (2013). Wnt signaling in triple negative breast cancer is associated with metastasis. BMC Cancer.

[16] Anastas JN, Moon RT (2013). WNT signalling pathways as therapeutic targets in cancer. Nat Rev Cancer.

[17] Incassati A, Chandramouli A, Eelkema R, Cowin P (2010). Key signaling nodes in mammary gland development and cancer: beta-catenin. Breast Cancer Res.

[18] Elsarraj HS, Hong Y, Valdez KE, Michaels W, Hook M, Smith WP, Chien J, Herschkowitz JI, Troester MA, Beck M, Inciardi M, Gatewood J, May L (2015). Expression profiling of *in vivo* ductal carcinoma in situ progression models identified B cell lymphoma-9 as a molecular driver of breast cancer invasion. Breast Cancer Res.

[19] Jenkins DE, Hornig YS, Oei Y, Dusich J, Purchio T (2005). Bioluminescent human breast cancer cell lines that permit rapid and sensitive *in vivo* detection of mammary tumors and multiple metastases in immune deficient mice. Breast Cancer Res.

[20] Revach OY, Geiger B (2014). The interplay between the proteolytic, invasive, and adhesive domains of invadopodia and their roles in cancer invasion. Cell Adh Migr.

[21] Huber F, Boire A, Lopez MP, Koenderink GH (2015). Cytoskeletal crosstalk: when three different personalities team up. Curr Opin Cell Biol.

[22] Jang GB, Kim JY, Cho SD, Park KS, Jung JY, Lee HY, Hong IS, Nam JS (2015). Blockade of Wnt/beta-catenin signaling suppresses breast cancer metastasis by inhibiting CSC-like phenotype. Sci Rep.

[23] Ridley AJ (2015). Rho GTPase signalling in cell migration. Curr Opin Cell Biol.

[24] Cadigan KM, Waterman ML (2012). TCF/LEFs and Wnt signaling in the nucleus. Cold Spring Harb Perspect Biol.

[25] Wotton D, Knoepfler PS, Laherty CD, Eisenman RN, Massague J (2001). The Smad transcriptional corepressor TGIF recruits mSin3. Cell Growth Differ.

[26] Zhang MZ, Ferrigno O, Wang Z, Ohnishi M, Prunier C, Levy L, Razzaque M, Horne WC, Romero D, Tzivion G, Colland F, Baron R, Atfi A (2015). TGIF governs a feed-forward network that empowers Wnt signaling to drive mammary tumorigenesis. Cancer Cell.

[27] Carey L, Winer E, Viale G, Cameron D, Gianni L (2010). Triple-negative breast cancer: disease entity or title of convenience?. Nat Rev Clin Oncol.

[28] Tam WL, Weinberg RA (2013). The epigenetics of epithelial-mesenchymal plasticity in cancer. Nat Med.

[29] Melotti A, Mas C, Kuciak M, Lorente-Trigos A, Borges I, Ruiz i Altaba A (2014). The river blindness drug Ivermectin and related macrocyclic lactones inhibit WNT-TCF pathway responses in human cancer. EMBO Mol Med.

[30] Jesse S, Koenig A, Ellenrieder V, Menke A (2010). Lef-1 isoforms regulate different target genes and reduce cellular adhesion. Int J Cancer.

[31] Liang J, Li Y, Daniels G, Sfanos K, De Marzo A, Wei J, Li X, Chen W, Wang J, Zhong X, Melamed J, Zhao J, Lee P (2015). LEF1 Targeting EMT in Prostate Cancer Invasion Is Regulated by miR-34a. Mol Cancer Res.

[32] Nguyen A, Rosner A, Milovanovic T, Hope C, Planutis K, Saha B, Chaiwun B, Lin F, Imam SA, Marsh JL, Holcombe RF (2005). Wnt pathway component LEF1 mediates tumor cell invasion and is expressed in human and murine breast cancers lacking ErbB2 (her-2/neu) overexpression. Int J Oncol.

[33] Nguyen DX, Chiang AC, Zhang XH, Kim JY, Kris MG, Ladanyi M, Gerald WL, Massague J (2009). WNT/TCF signaling through LEF1 and HOXB9 mediates lung adenocarcinoma metastasis. Cell.

[34] Huang FI, Chen YL, Chang CN, Yuan RH, Jeng YM (2012). Hepatocyte growth factor activates Wnt pathway by transcriptional activation of LEF1 to facilitate tumor invasion. Carcinogenesis.

[35] Chen C, Cao F, Bai L, Liu Y, Xie J, Wang W, Si Q, Yang J, Chang A, Liu D, Liu D, Chuang TH, Xiang R (2015). IKKbeta Enforces a LIN28B/TCF7L2 Positive Feedback Loop That Promotes Cancer Cell Stemness and Metastasis. Cancer Res.

[36] Hsieh TH, Hsu CY, Tsai CF, Chiu CC, Liang SS, Wang TN, Kuo PL, Long CY, Tsai EM (2016). A novel cell-penetrating peptide suppresses breast tumorigenesis by inhibiting beta-catenin/LEF-1 signaling. Sci Rep.

[37] Ravindranath A, Yuen HF, Chan KK, Grills C, Fennell DA, Lappin TR, El-Tanani M (2011). Wnt-beta-catenin-Tcf-4 signalling-modulated invasiveness is dependent on osteopontin expression in breast cancer. Br J Cancer.

[38] Lan F, Yue X, Han L, Shi Z, Yang Y, Pu P, Yao Z, Kang C (2012). Genome-wide identification of TCF7L2/TCF4 target miRNAs reveals a role for miR-21 in Wnt-driven epithelial cancer. Int J Oncol.

[39] Yu Y, Wu J, Wang Y, Zhao T, Ma B, Liu Y, Fang W, Zhu WG, Zhang H (2012). Kindlin 2 forms a transcriptional complex with beta-catenin and TCF4 to enhance Wnt signalling. EMBO Rep.

[40] Xiang G, Yi Y, Weiwei H, Weiming W (2015). TGIF1 promoted the growth and migration of cancer cells in nonsmall cell lung cancer. Tumour Biol.

[41] Huang HS, Liu ZM, Chen PC, Tseng HY, Yeh BW (2012). TG-interacting factor-induced superoxide production from NADPH oxidase contributes to the migration/invasion of urothelial carcinoma. Free Radic Biol Med.

[42] Yeh BW, Wu WJ, Li WM, Li CC, Huang CN, Kang WY, Liu ZM, Huang HS (2012). Overexpression of TG-interacting factor is associated with worse prognosis in upper urinary tract urothelial carcinoma. Am J Pathol.

[43] Cheng J, Blum R, Bowman C, Hu D, Shilatifard A, Shen S, Dynlacht BD (2014). A role for H3K4 monomethylation in gene repression and partitioning of chromatin readers. Mol Cell.

[44] Solaimani P, Wang F, Hankinson O (2014). SIN3A, generally regarded as a transcriptional repressor, is required for induction of gene transcription by the aryl hydrocarbon receptor. J Biol Chem.

[45] Reynolds N, O'Shaughnessy A, Hendrich B (2013). Transcriptional repressors: multifaceted regulators of gene expression. Development.

[46] Varier RA, Carrillo de Santa Pau E, van der Groep P, Lindeboom RG, Matarese F, Mensinga A, Smits AH, Edupuganti RR, Baltissen MP, Jansen PW, Ter Hoeve N, van Weely DR, Poser I (2016). Recruitment of the mammalian histone modifying EMSY complex to target genes is regulated by ZNF131. J Biol Chem.

[47] Hamid R, Brandt SJ (2009). Transforming growth-interacting factor (TGIF) regulates proliferation and differentiation of human myeloid leukemia cells. Mol Oncol.

[48] Lee BK, Shen W, Lee J, Rhee C, Chung H, Kim KY, Park IH, Kim J (2015). Tgif1 Counterbalances the Activity of Core Pluripotency Factors in Mouse Embryonic Stem Cells. Cell Rep.

[49] Zang C, Schones DE, Zeng C, Cui K, Zhao K, Peng W (2009). A clustering approach for identification of enriched domains from histone modification ChIP-Seq data. Bioinformatics.

