# Supplementary material for: Targeted interference of SIN3A-TGIF1 function by SID decoy treatment inhibits Wnt signaling and invasion in triple negative breast cancer cells
Source: Oncotarget. 2016 Aug 19;8(51):88421–36. doi: 10.18632/oncotarget.11381 (PMC5687616; doi:10.18632/oncotarget.11381)
Supplement: Supplementary file 1 [file oncotarget-08-88421-s001.pdf]

Targeted interference of SIN3A-TGIF1 function by SID decoy treatment inhibits Wnt signaling and invasion in triple negative breast cancer cells

SUPPLEMENTAL FIGURES AND TABLES

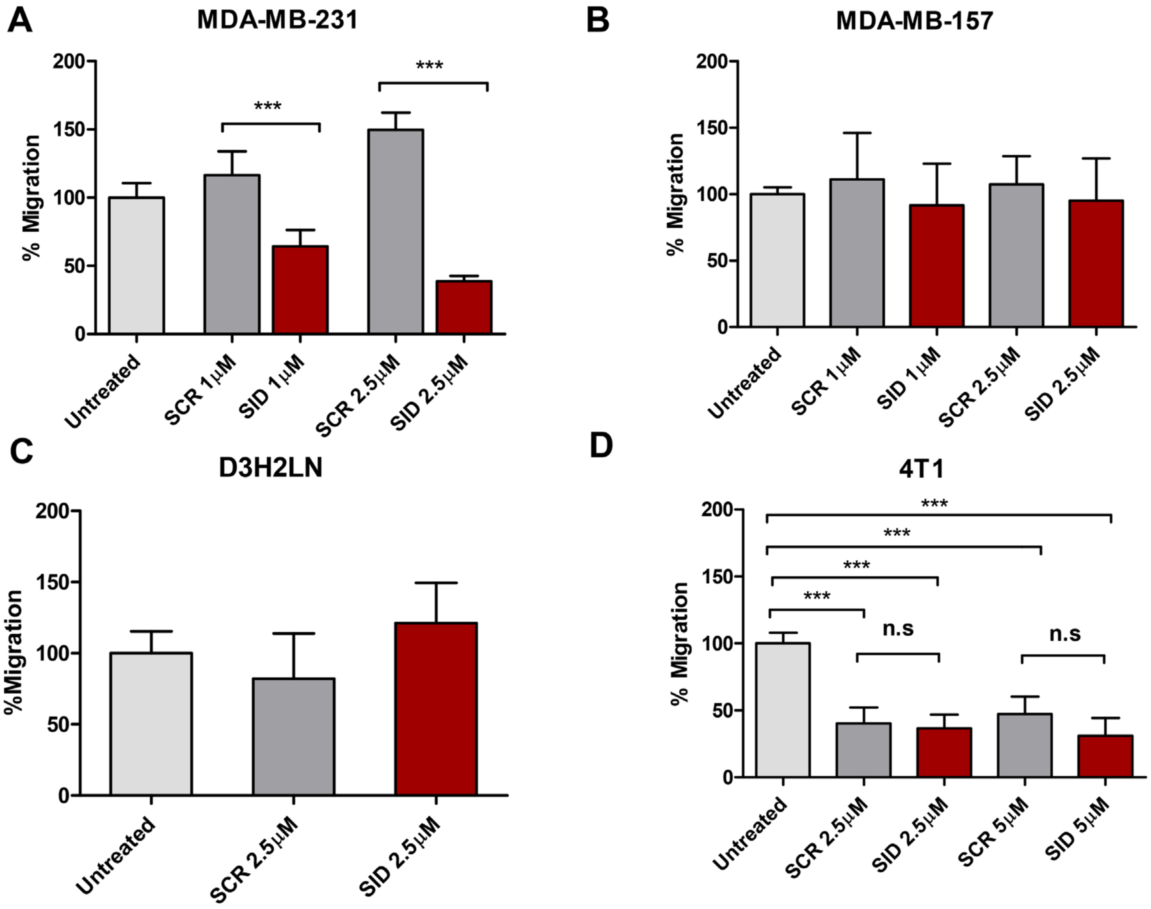

Supplementary Figure S1: Treatment with SID decoy peptide does not significantly change transwell migration of TNBC cells. A-D. Transwell migration assays were performed with MDA-MB-231 (A), MDA-MB-157 (B), D3H2LN (C), or 4T1 (D) cells that were incubated with scramble (SCR; 1, 2.5 or 5μM) or SID peptide (SID; 1, 2.5, 5 μM). The percentage of migration for SCR and SID peptide treated cells was calculated by normalizing the number of cells invaded per filter in comparison to of untreated control. (A;***P<0.0001, B; P=0.9840, C; P=0.5825, D; ***P=0.0006, One-way ANOVA followed by Tukey's multiple comparison test, error bars, mean ± SEM, *n.s- not significant)

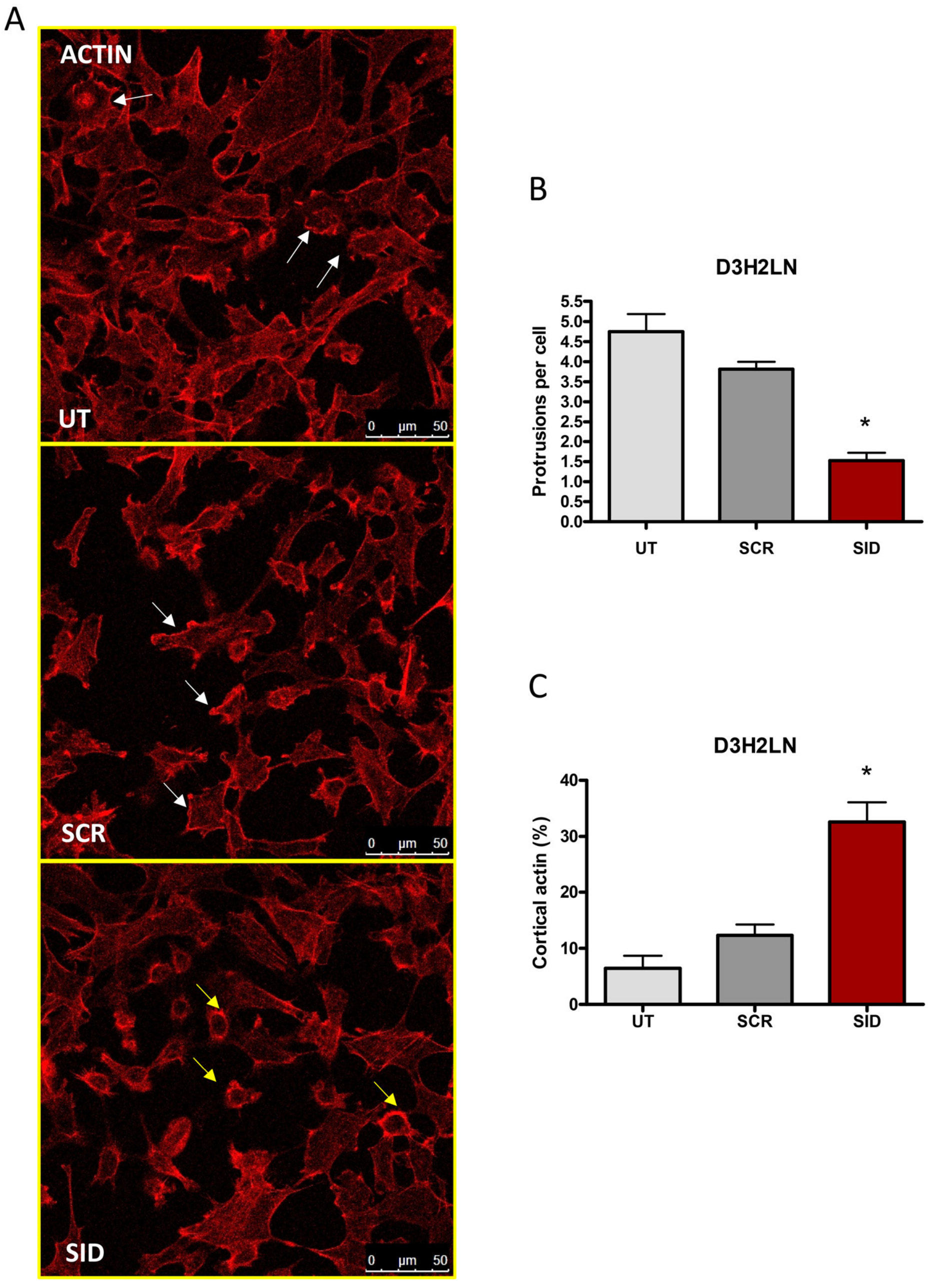

Supplementary Figure S2: SID peptide treatment alters actin cytoskeletal organization in D3H2LN TNBC cells. A. D3H2LN cells that were either untreated or treated with 2.5µM SCR or SID peptide for 24 hours were stained with rhodamine phalloidin dye. **B.** The number of protrusions (marked with white arrows as seen in Figure 1A) per cell was quantified (*P<0.0001, One-way ANOVA, error bars, mean ± SEM). **C.** The percentage of cells with cortical actin (marked in yellow arrow as seen in Figure 1A) was quantified (*P<0.0001, One-way ANOVA, error bars, mean ± SEM).

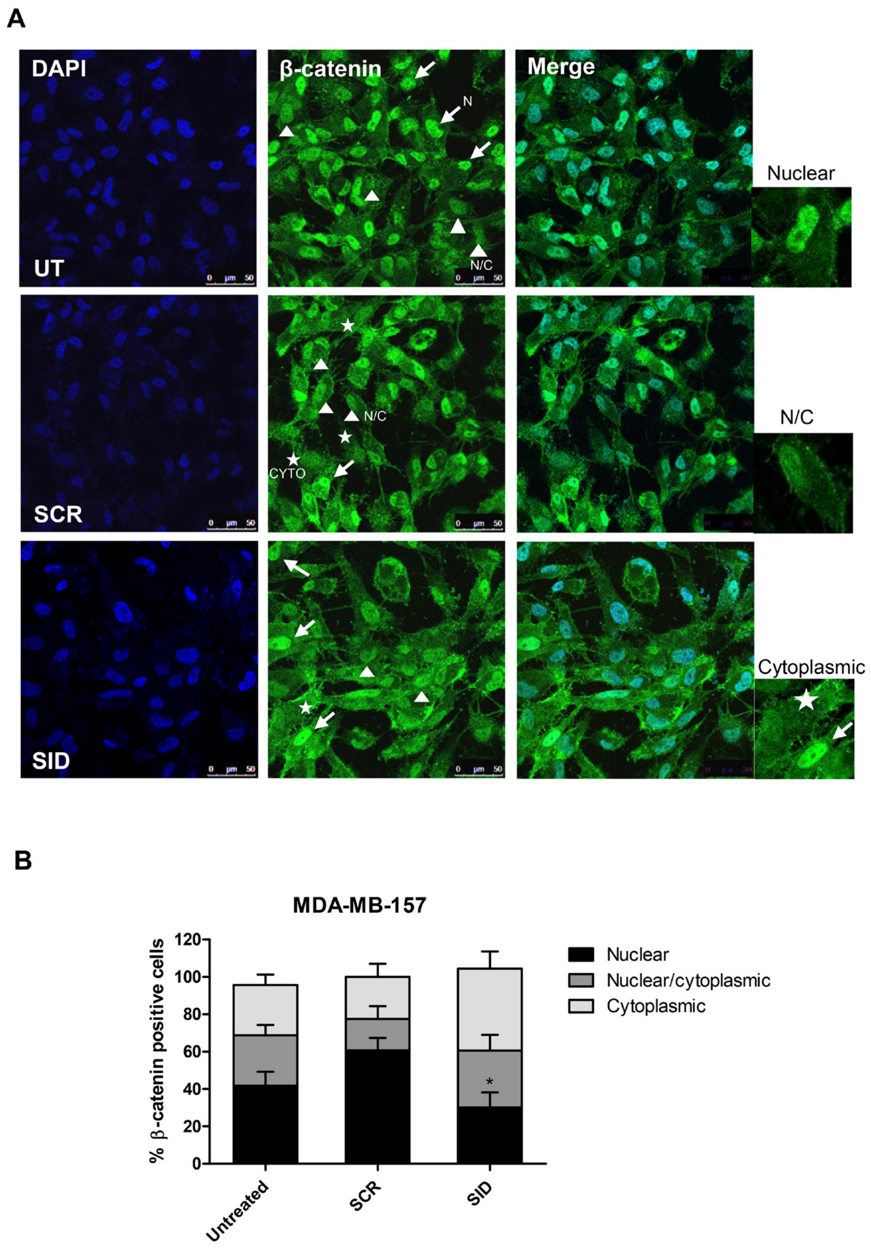

Supplementary Figure S3: Nuclear, nuclear/cytoplasmic, cytoplasmic localization of β-catenin in MDA-MB-157 cells that were untreated or treated with SCR or SID peptide (2.5μM) for 24 hours. A. Immunofluorescence staining of β-catenin in MDA-MB-157 cells untreated or treated with SCR or SID peptide (2.5μM, 24 hours). Arrow points to the nuclear (N), triangle (▲) points to nuclear/cytoplasmic (N/C), star- cytoplasmic (C) **B.** The quantification of % cells demonstrating nuclear, nuclear/cytoplasmic, and cytoplasmic β-catenin localization (nuclear; * P=0.0336, One-way ANOVA, SID vs. SCR, error bar, mean ± SEM)

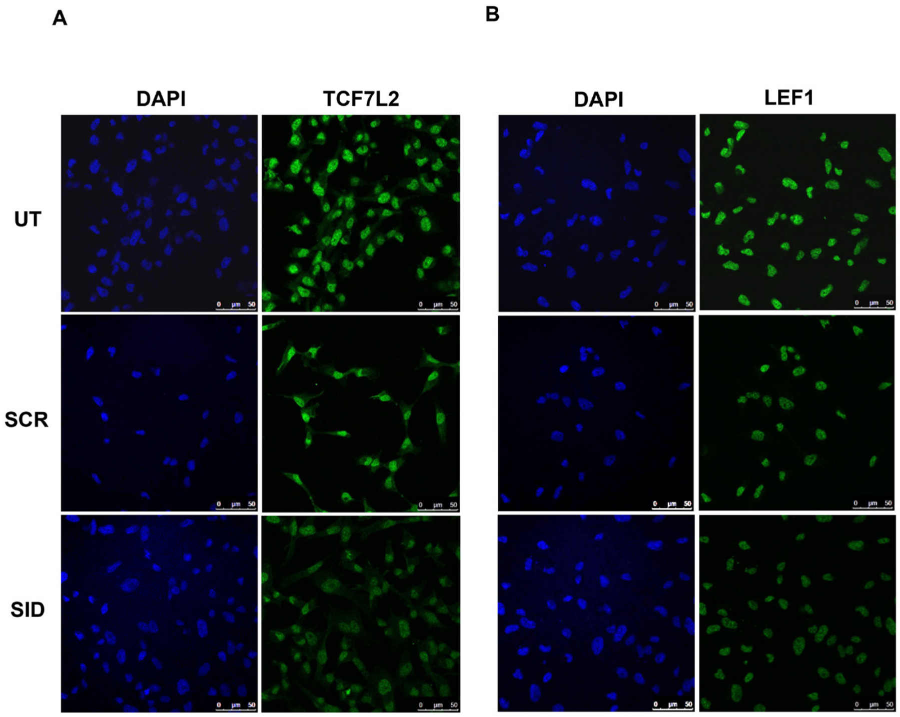

Supplementary Figure S4: Subcellular localization of TCF7L2 and LEF1 in D3H2LN cells untreated or treated with SCR or SID peptide (2.5µM, 24 hours).

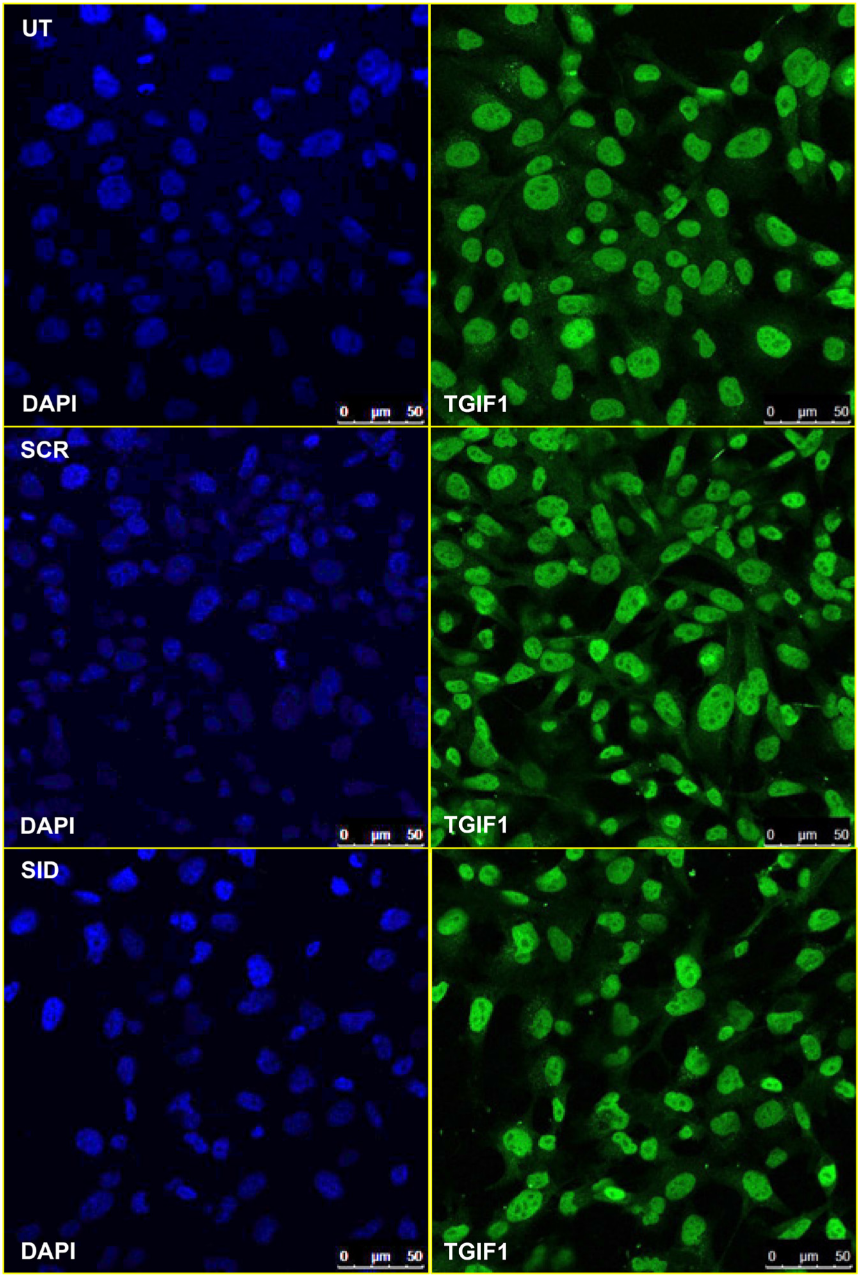

Supplementary Figure S5: Subcellular localization of TGIF1 in D3H2LN cells that were untreated or treated with 2.5µM SCR or SID peptide for 24 hours.

Supplementary Table S1: primer sequences used for qPCR analysis

	sense	antisense
**MT1-MMP**	CACTGCCTACGAGAGGAAGG	TTGGGGTACTCGCTATCCAC
**MMP9**	CCCTCAGTGAAGCGGTACAT	TTGACAGCGACAAGAAGTGG
**LEF1**	TCAAACAGGAACATCCCCAC	TGAGGTCTTTTTGGCTCCTG
**CTNNB1**	GCTACTCAAGCTGATTTGATGGA	GGTAGTGGCACCAGAATGGATT
**PLAU**	GGGTCGCTCAAGGCTTAACT	CGTGTCAGCGCTGTAGTCC
**RPL30**	GACAAGGCAAAGCGAAATTG	GTATTTTCCGCATGCTGTGC

Supplementary File 1: Affymatrix gene microarray for MDA-MB-231 cells (2.5µM SID vs SCR peptide treatment for 24 hours)

See Supplementary File 1

Supplementary File 2: Ingenuity canonical pathway analysis for MDA-MB-231 cells (2.5µM SID vs SCR peptide treatment for 24 hours)

See Supplementary File 2

Supplementary File 3: The list of genes that predict CTNNB1 upstream regulator to be inhibited (by IPA analysis)

See Supplementary File 3
